# Trap-state mapping to model GaN transistors dynamic performance

**DOI:** 10.1038/s41598-022-05830-7

**Published:** 2022-02-02

**Authors:** Nicola Modolo, Carlo De Santi, Andrea Minetto, Luca Sayadi, Gerhard Prechtl, Gaudenzio Meneghesso, Enrico Zanoni, Matteo Meneghini

**Affiliations:** 1 Department of Information Engineering, University of Padova, via Gradenigo 6/B, 35131 Padova, Italy; 2 Infineon Technologies Austria, Siemensstraße 2, 9500 Villach, Austria

**Keywords:** Electrical and electronic engineering, Electronics, photonics and device physics

## Abstract

Trapping phenomena degrade the dynamic performance of wide-bandgap transistors. However, the identification of the related traps is challenging, especially in presence of non-ideal defects. In this paper, we propose a novel methodology (trap-state mapping) to extract trap parameters, based on the mathematical study of stretched exponential recovery kinetics. To demonstrate the effectiveness of the approach, we use it to identify the properties of traps in AlGaN/GaN transistors, submitted to hot-electron stress. After describing the mathematical framework, we demonstrate that the proposed methodology can univocally describe the properties of the distribution of trap states. In addition, to prove the validity and the usefulness of the model, the trap properties extracted mathematically are used as input for TCAD simulations. The results obtained by TCAD closely match the experimental transient curves, thus confirming the accuracy of the trap-state mapping procedure. This methodology can be adopted also on other technologies, thus constituting a universal approach for the analysis of multiexponential trapping kinetics.

## Introduction

Charge-trapping is a critical factor in determining the dynamic performance of electronic devices (transistors and diodes). This is relevant for conventional silicon transistors, but more critical for wide bandgap semiconductor devices, which can operate at much higher frequencies and electric fields. As an example, GaN transistors used in power conversion can reach electric fields above 2 MV/cm^1^, and operate at frequencies above 1–10 MHz and very low duty cycles^2^, with dV/dt in the range of 10–100 V/ns^3^.

Such dynamic performance can be exploited only through a minimization of the trapping phenomena, which originate from defects located in the semiconductor material, at the heterointerfaces, or at the surface of the devices.

For an accurate TCAD (Technology computer-aided design) modeling of device characteristics, a reliable technique for the characterization of trap-states must be developed.

Such characterization is normally carried out through current transient measurements^4^, and trap parameters are typically extracted under the oversimplistic approximation that defects are ideal (thus resulting in single-exponential current transients) or have a Gaussian distribution of time constants^5–10^.

However, this is an empirical approach, that does not consider the physical origin of traps: (i) surface or interface states may have a broad distribution of energies and cross sections, thus resulting in turn-on transients that substantially deviate from ideal exponentials, and (ii) a Gaussian distribution relies in the (always false) approximation that the single component kinetic is a step-function instead of an exponential decay.

The question is therefore, is it possible to define a methodology to quantitatively extract trap parameters, in presence of non-ideal (non-exponential) de-trapping processes? In this article, we address this issue by proposing a novel methodology (Trap-State Mapping), that provides a quantitative description of the activation energy and cross section distribution of traps.

Before describing our approach, to give a bit of background, we remind that if carriers are trapped at discrete energy levels in the bandgap, an almost ideal exponential recovery is observed, which is described by the simple exponential decay function^11^:

1 $$ \frac{n\left( t \right)}{{N_{t} }} = \exp \left( { - \frac{t}{{\tau_{0} }}} \right) $$

where $N_{t}$ is the density of filled traps after a certain electrical stress, $n\left( t \right)$ is the density of trapped electrons, and $\tau_{0}$ is the time constant of the detrapping process. This equation comes from the solution of the linear first order differential equation:

2 $$ \frac{dn\left( t \right)}{{dt}} = - \frac{n\left( t \right)}{{\tau_{0} }}. $$

On the other hand, especially for non-ideal interface and surface traps, the pure exponential relaxation rarely forms in nature. In such cases the relaxation kinetics show stretched-exponential trends^12^, that is often explained by considering a distribution of activation energies and cross sections for the individual traps involved^13–15^.

Considering that (i) surface and interfaces play a key-role in determining the dynamic performance of wide bandgap HEMT devices and, (ii) de-trapping from surface states is characterized by a distribution of activation energies and capture cross sections, which results in a heavily-spread time-constant spectrum, the mathematical description of how these competing mechanisms interact with each other during the recovery assumes great importance.

A commonly used approach for extracting interface/surface trap parameters is based on capacitance^14,16^ or conductance^17^ measurements on capacitors, to extract the interface state density (D_it_). However, this requires specific test structures, whose properties and processing parameters may deviate from those of final devices.

With this paper, we want to overcome this issue, by proposing an effective and reliable methodology for extracting the interface trap properties (activation energy and capture cross section distribution) starting from the analysis of the stretched exponential transients measured on real transistors.

We start by developing the mathematical framework for the analysis of stretched exponential decay functions, and we show that the trap parameters can be univocally extracted through multi-exponential analysis. Specifically, we consider that the density of occupied traps $n\left( t \right)$ during relaxation is described as:

3 $$ f\left( t \right) = \frac{n\left( t \right)}{{N_{T} }} = \mathop \sum \limits_{i = 1}^{k} \frac{{n_{i} \left( t \right)}}{{N_{T} }} = \mathop \sum \limits_{i = 1}^{k} A_{i} \cdot \exp \left( { - \frac{t}{{\tau_{i} }}} \right) $$

where $\tau_{1} , \tau_{2} , \ldots \tau_{k}$ is a finite set of discrete time constants, $N_{T} = N_{1} + N_{2} + \cdots + N_{k}$ is the total density of filled traps and, $A_{i} = N_{i} /N_{T}$ is the normalized amplitude of each exponential decay function associated to the relative time constant $\tau_{i}$. Then, we describe a methodology for the identification of the trap parameters, univocally described by the values of $A_{i}$ and $\tau_{i}$. This procedure is made difficult by the fact that we are dealing with a series of non-linear and non-orthogonal functions.

The developed mathematical framework is then used to interpret the relaxation kinetics of AlGaN/GaN HEMTs submitted to hot electron stress. On top of this, for the first time, we demonstrate how, from the extracted time constant distributions at different temperatures, the trap states properties can be univocally described. This is achieved by adapting the Arrhenius law in case of distributed trap levels for the effective extraction of the activation energy $\left( {E_{a,i} } \right)$and capture cross section and $\left( {\sigma_{c,i} } \right)$ distributions.

Finally, the extracted trap parameters are used as input for TCAD simulations: we show that the results obtained by TCAD closely reproduce the experimental ones, thus demonstrating the effectiveness of the presented approach in quantitatively mapping the properties of trap states.

The results described here are referred to AlGaN/GaN HEMTs; however, the methodology developed here offers a universal approach to extrapolate the time-constant spectrum profile in heavily stretched exponential decays, that can be used also for investigating different technologies and different physical processes.

## Theory of trap-state mapping via stretched-exponential functions

A quantitative trap-state mapping requires the extraction of the distribution of activation energies and cross sections for a set of states, which result in stretched-exponential transient for a transistor. In the past decades, many mathematical methods have been developed in order to study the nature of stretched exponential functions^18–24^. This is because of its exceptionally wide range of application throughout many fields of science^18^.

These methods rely upon the approach formulated in 1959 by Gardner et al*.*^21^, which is based on the inversion of the Laplace integral equation by a Fourier transforms method. A main concern in this approach arises from the fact that the experimental data is often noisy and truncated, so an accurate extraction of the parameters may be prevented.

In this section, we propose two approaches to identify the normalized amplitude of the individual exponential components (${A}_{i}$) and the related time constants (${\tau }_{i}$) that constitute a stretched exponential transient. The methodologies can be used whenever the quality of the experimental data permits a fit with the multiexponential decay function $f(t)$.

### Asymptotic approach

We start considering that the function $f\left( t \right)$ in Eq. (3) is in the form of a Dirichlet series, which may be generalized in a continuum of amplitudes $A_{i} = g\left( \nu \right)d\nu$ as:

4 $$ f\left( t \right) = \mathop \sum \limits_{i = 1}^{k} A_{i} \cdot \exp \left( { - t\nu_{i} } \right)\sim \mathop \smallint \limits_{0}^{\infty } g\left( \nu \right) \cdot {\text{exp}}\left( { - t\nu } \right) d\nu $$

where $\nu_{i} = \tau_{i}^{ - 1}$ and $g\left( \nu \right)$ is the probability density function such that:

5 $$ \mathop \smallint \limits_{0}^{\infty } g\left( \nu \right) d\nu = 1. $$

Therefore, the extraction of the $g\left( \nu \right)$ profile is a necessary step to find the time-constant distribution of the experimentally determined data.

A common methodology, when studying the relaxation kinetics of multiexponential functions, consists of fitting the experimental data with the semi-empirical stretched exponential function^22^, and then posing the equality:

6 $$ f\left( t \right) = \mathop \smallint \limits_{0}^{\infty } g\left( \nu \right) \cdot {\text{exp}}\left( { - t\nu } \right) d\nu = \exp \left( { - \frac{t}{{\tau_{0} }}} \right)^{\beta } $$

where the stretching exponent $\beta$ is a semi-empirical parameter in the range (0,1]. In this specific case, the $g\left( \nu \right)$ profile can be computed by expanding the stretched exponential function in Taylor series^22,23^ as:

7 $$ g\left( \nu \right) = - \frac{1}{\pi \nu } \cdot \mathop \sum \limits_{n = 1}^{\infty } \frac{{\left( { - 1} \right)^{n} }}{n!} \cdot \frac{{\sin \left( {n\pi \beta } \right)}}{{\left( {\nu t} \right)^{n\beta } }}\Gamma \left( {n\beta + 1} \right) $$

where $\Gamma$ is the gamma function^24^. This function does not have a closed solution. However, for the special values $\beta = 1/2$ and $\beta = 1$, Eq. (7) can be written as:

8 $$ g\left( \nu \right) = \delta \left( {v - \frac{1}{{\tau_{0} }}} \right);\;\beta = 1 $$

9 $$ g\left( \nu \right) = \frac{1}{{2\sqrt {\pi \tau_{0} \nu^{3} } }} \cdot \exp \left( { - \frac{1}{{4\tau_{0} \nu }}} \right).\beta = \frac{1}{2} $$

where $\delta$ is the Dirac function centered in $\tau_{0}^{ - 1}$. Recently, Johnston calculated the analytical function for $\beta = 1/3$ and $\beta = 2/3$^25^. However, for a general value of $\beta$ the function $g\left( \nu \right)$ can be obtained in asymptotic form using the saddle point method of integration in Eq. (6) as follows^23^:

10 $$ g\left( v \right) = \frac{ab}{{\sqrt {2\pi \beta } }}\left( {\nu b} \right)^{{ - \left( {1 + \frac{a}{2}} \right)}} \exp \left( { - \left( {\frac{1}{\nu b}} \right)^{a} } \right) $$

where $$a = \frac{\beta }{1 - \beta };\;b = \frac{{\tau_{0} }}{{\beta \left( {1 - \beta } \right)^{1/a} }}.$$

**Figure 1:**
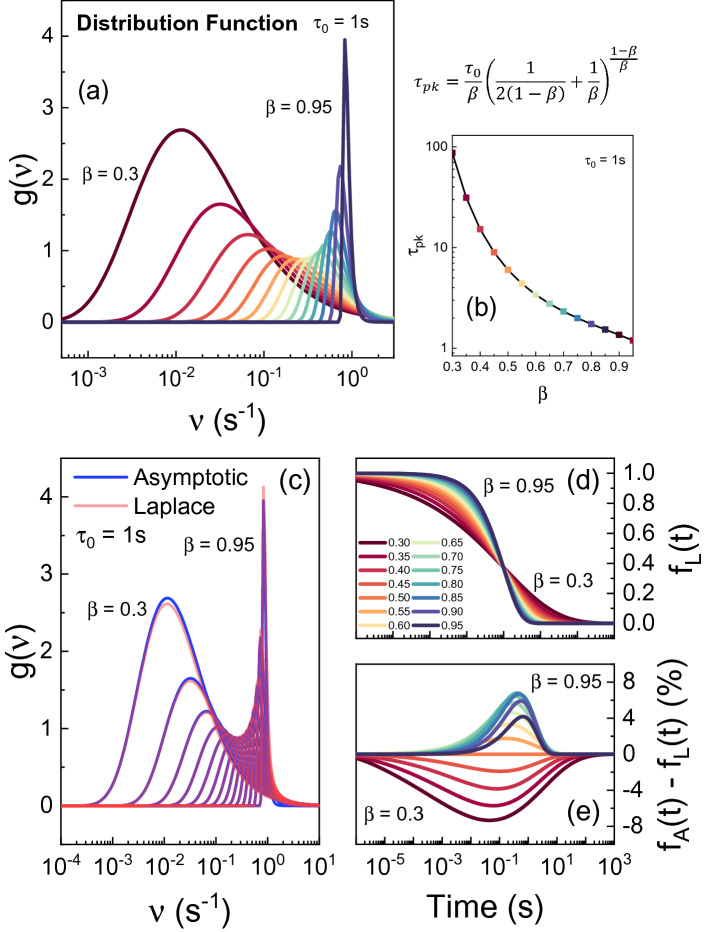
(**a**) Distribution function $g\left( v \right)$ in Eq. (10) for different values of $\beta$ from 0.3 to 0.95. (**b**) Dependency between $\tau_{pk} /\tau_{0}$ and $\beta$. Notably, the difference between $\tau_{pk}$ and $\tau_{0}$ reaches several orders of magnitude for low values of $\beta$. (**c**) Comparison between the distribution function $g\left( v \right)$ using the asymptotic (red) and inverse Laplace (blue) approach. (**d**) $f\left( t \right)$ calculated by integrating $g\left( \nu \right)$ numerically calculated with the inverse Laplace transform varying $\beta$ from 0.3 to 0.95. (**e**) Relative error introduced using the asymptotic extraction (saddle point method) in respect to the inverse Laplace transform.

It can be noted that, for $\beta = 1/2$, Eq. (10) coincides with Eq. (9). Finally, Fig. 1a shows the probability density function $g\left( v \right)$ vs $\nu = \tau^{ - 1}$, illustrated for some values of $\beta$ with $\tau_{0} = 1$ s. If $\beta \to 1$, then $g\left( v \right)$ is described by the Dirac function centered in $\tau_{0}$. Furthermore, by decreasing $\beta$, the peak $\tau_{pk}$ of the probability density function changes and becomes different from $\tau_{0}$. The dependency between the peak $\tau_{pk}$ and $\beta$ can be calculated from the derivative of Eq. (10) as:

11 $$ \tau_{pk} = \frac{dg\left( v \right)}{{d\beta }} = \frac{{\tau_{0} }}{\beta } \cdot \left( {\frac{1}{{2\left( {1 - \beta } \right)}} + \frac{1}{\beta }} \right)^{{\frac{1 - \beta }{\beta }}} . $$

Figure 1b shows the dependency between $\tau_{pk} /\tau_{0}$ and $\beta$. On one hand, for high values of $\beta$ (ideal exponential decay), $\tau_{pk}$ and $\tau_{0}$ coincides. On the other hand, for low values of $\beta$ (stretched exponential) the ratio between the two increases significantly leading to counterintuitive results. For example, if $\beta = 0.3$, $\tau_{pk}$ is two order of magnitude larger than $\tau_{0}$ meaning that the extracted $\tau_{0}$ and the peak of the time-constant spectrum do not coincide.

To conclude, the analytical approach presents some main advantages such as a closed analytical function (easy to implement) and a good accuracy. However, an asymptotic solution is not the most accurate solution (see also next section) and this approach only works with a single stretched exponential centered in $\tau_{0}$. Therefore, in the next section, the inverse Laplace transform approach is proposed.

### Inverse Laplace transform approach

A second approach to extract the probability distribution function, consists in recognizing that the integral of Eq. (4) has Laplacian form. Therefore, the stretched exponential function $f\left( t \right)$ is the Laplace transform of $g\left( \nu \right)$.

Using an analytical expression, such as the one in Eq. (6) to define $f\left( t \right)$, with $\beta$ and $\tau_{0}$ taken from the experimental data, it is possible to extract $g\left( \nu \right)$ by means of inverse Laplace Transform, solving the following equation:

12 $$ g\left( \nu \right) = L^{ - 1} \left\{ {f\left( t \right)} \right\} = L^{ - 1} \left\{ {{\text{exp}}\left( { - \left( {\frac{t}{{\tau_{0} }}} \right)^{\beta } } \right)} \right\}. $$

It can be recognized that, if $\beta = 1$, Eq. (12) has closed solution $g\left( \nu \right) = \delta \left( {v - \tau_{0}^{ - 1} } \right)$. Alternatively, the inversion may be computed numerically. In Fig. 1c the numerical solution (blue) and the asymptotic solution (red) are compared. The result shows that the accuracy obtained using the saddle point method is quite good, validating Eq. (11) for the $\tau_{pk}$ extraction. However, as can be seen in Fig. 1d,e, by integrating $g\left( \nu \right)$ in Eq. (6) the error introduced by the asymptotic approach is not negligible. Finally, the amplitude $A_{i}$ of each component can be extracted as $A_{i} = g\left( \nu \right)d\nu$.

**Figure 2:**
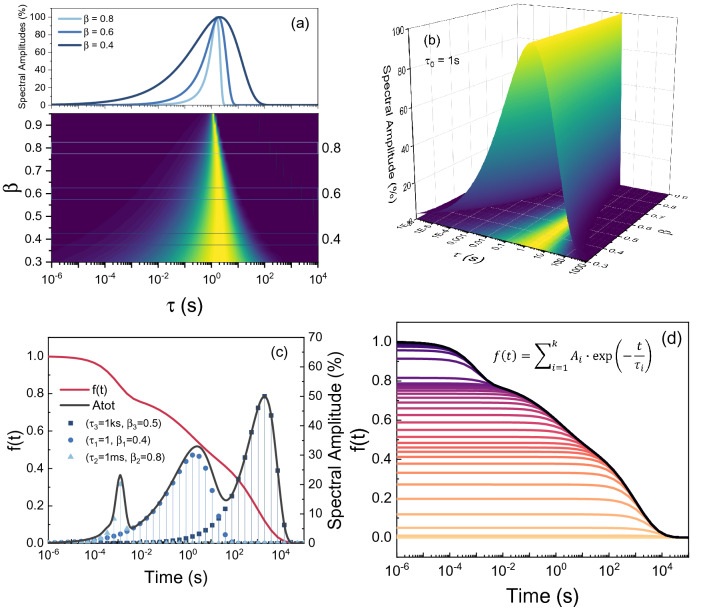
(**a**) Contour map of the $A_{i}$ distribution for different values of $\beta$ with $\tau_{0} = 1$ s. (**b**) 3D plot of the $A_{i}$ distribution of time constants for a fixed $\tau_{0} = 1$ s and different stretching coefficient. (**c**) $f\left( t \right)$ defined as three stretched exponential functions. (**d**) The sum of each exponential decay component ($A_{i}$) returns the stretched exponential behavior.

Figure 2 shows the (a) contour plot and (b) 3D map of the $A_{i}$ distribution for different values of $\beta$ with $\tau_{0} = 1$ s. A high $\beta$ (ideal exponential decay) tends to the Dirac function. On the contrary, a low $\beta$ leads to a very broad distribution.

Since $\nu = \tau^{ - 1}$, the $A_{i}$ distribution can be plotted together with the stretched exponential function by simply inverting the x-axis. The superimposition between the $A_{i}$ distribution and its relative stretched exponential is shown in Fig. 2c where, $f\left( t \right)$ is defined as the sum of three stretched exponential functions. Each $A_{i}$ is plotted as a delta function centered in its respective time-constant $\tau_{i}$.

To verify the result, the extracted $A_{i}$ distribution is used in Eq. (3) which physically describes the relaxation to the ground state occurring through many competing channels. Figure 2d shows that the summation of each exponential decay multiplied for the extracted $A_{i}$ returns the correct $f\left( t \right)$ even for a multiple stretched exponential decay, thus validating the extraction procedure.

## Experimental trap-state mapping on GaN-based transistors

The mathematical framework presented in the previous section helps to understand the physical nature of the stretched exponential, which may be useful for a broad range of applications.

Based on these considerations, we demonstrate the applicability of the developed mathematical framework to the trap-state mapping in in normally-off p-GaN HEMTs after semi-on stress. During this stress condition, the device must sustain a simultaneously high drain-to-gate electric field and carrier density^26^. The carriers accelerated by the high field are described as “hot”, and their excess kinetic energy may favor trapping effects that are not reachable under normal (off-state) conditions.

In our previous works we demonstrated that the hot-electrons injection mainly involves surface trapping^27^. To summarize our results, we observed that the trapping kinetics follows a logarithmic trend, thus reconducting it to an inhibition effect originated from the Coulombic repulsion between a filled trap and a free electron injected to the surface^28^.

In GaN HEMTs, the charge trapped at the surface depletes the 2DEG channel and can be quantified by monitoring the dynamic-$R_{on}$ increase. Therefore, the measurements were done in the linear region since, for a given voltage, current is inversely proportional to the dynamic-$R_{on}$. From previous investigations, we observed that after 10 µs of semi-on stress the very fast hot electron trapping already induced a significant current collapse on the device while the off-state contribution is negligible. Consequently, the test is performed by monitoring, through fast I_D_V_G,_ the DCT of the device under test (DUT) biased in the linear region at $V_{ON} = \left( {V_{G} , V_{D} } \right) = \left( {4, 0.4} \right)$ V, after a filling time $t_{fill} = 10$ µs at $V_{fill} = \left( {2, 50} \right)$ V, to inject the hot electrons to the surface.

**Figure 3:**
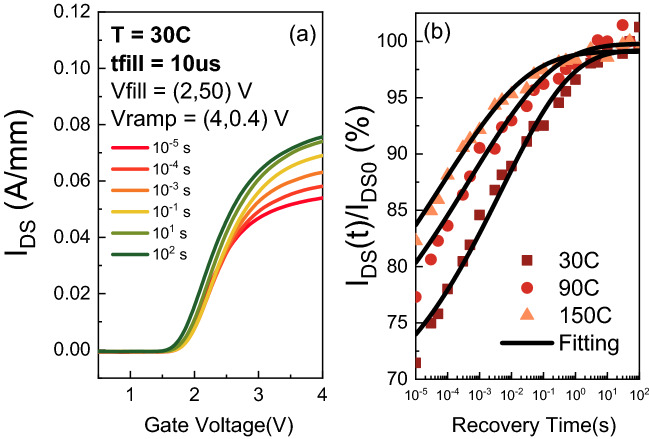
(**a**) Logarithmically spaced I_D_V_G_ at $V_{DS} = 0.4$ V after $t_{fill} = 10$ µs at $V_{fill} = \left( {2, 50} \right)$ V. (**b**) Normalized drain current transient at different temperatures and $V_{ON} = \left( {4, 0.4} \right)$ V. The recovery is very slow compared to the fill time.

Figure 3a shows the I_D_V_G_ taken at different moments during the recovery phase at room temperature. The red curve represents the transfer characteristics taken immediately after the stress. From a qualitative perspective, the 10 µs hot-electron injection in the surface induces a considerable current collapse in the device (from 80 mA/mm to 50 mA/mm at $V_{G} = 4$ V).

Furthermore, despite the short filling time, the recovery process is very slow. To obtain a quantitative evaluation of the relaxation kinetics, Fig. 3b shows the drain current transient of the DUT at $V_{ON} = \left( {4, 0.4} \right)$ V. The measurement has been repeated at different temperatures: T = (30, 90, 150) °C.

The detrapping kinetics are fitted with Eq. (6) and show a strongly stretched-exponential behavior. The set of parameters $A_{T}$, $\beta_{T}$ and $\tau_{T}$ extracted at each temperature is reported in Table 1.

**Table 1:** *f*(*t*) fitting parameter.

	30 °C	90 °C	150 °C
*A*_*T*_	0.38	0.37	0.37
*β*_*T*_	0.198	0.174	0.159
*τ*_*T*_	4.72 × 10^−3^ s	4.67 × 10^−4^ s	6.06 × 10^−5^ s

**Figure 4:**
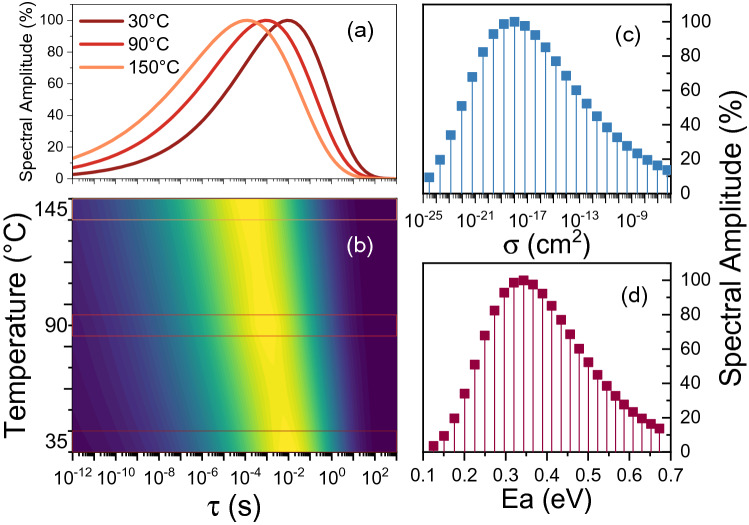
(**a–b**) $A_{i}$ distribution mapped extracted from the experimental data and mapper at different temperatures; (**c**) Distribution of activation energies, and (**d**) capture cross sections in the surface of the device under test.

Figure 4a,b shows the contour map of the time constant distribution numerically extracted by solving the Inverse Laplace Transform function from the experimental data and mapped at different temperatures. Here, the $g\left( \nu \right)$ profile extraction is obtained by Euler numerical inversion^29^ from the fitted function $f\left( t \right)$. Alternatively, for a direct extraction from the measured $I_{DS} \left( t \right)$, other numerical strategies require the solution of a first kind Fredholm integral equation where the kernel is the decaying exponential function^30,31^. The solution of this ill-posed inverse problem is extremely sensitive to noise; however, substantial improvements can be obtained by the application of Tikhonov regularization^32^–^34^.

**Figure 5:**
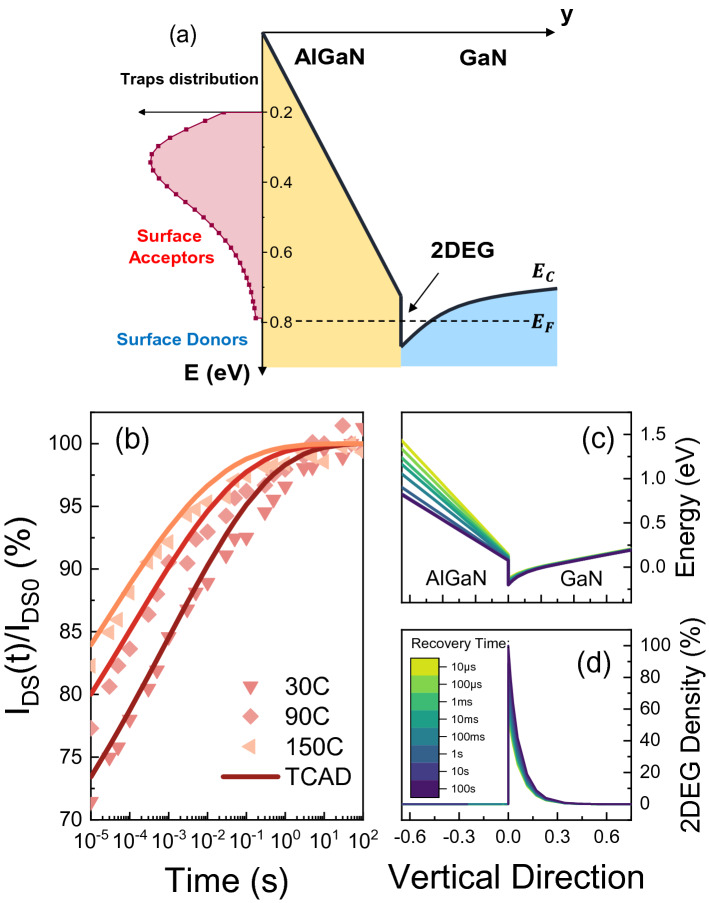
(**a**) Trap distribution at the passivation/AlGaN interface implemented in TCAD structure. The activation energies and capture cross sections are defined according to the distributions obtained via trap-state mapping. (**b**) TCAD simulation of the recovery transient repeated at different temperatures, and (**c–d**) Vertical cross section of the dynamic behavior of the conduction band during the recovery along with the repopulation of the 2DEG Density.

The increase in temperature affects the profile by changing both the peak position and the width of the curve. Considering that the physical nature of the stretched behavior can be attributed to the intrinsic defectiveness of the surface region, characterized by a distribution of activation energies ($E_{a}$) and capture cross sections ($\sigma_{c}$), then the mathematical description developed in the previous section can give an extraordinary insight on the time constant distribution. This is because, in semiconductor physics, time constants are strictly linked to the activation energy and capture cross section of a trap level as^35^:

13 $$ \ln \left( {\tau T^{2} } \right) = \frac{{E_{a} }}{kT} + \ln \left( {\frac{{h^{3} }}{{2\sqrt 3 \left( {2\pi } \right)^{\frac{3}{2}} m_{r} m_{0} k^{2} g\sigma_{c} }}} \right) $$

where $T$ is the temperature, $h$ is the Planck constant, $k$ is the Boltzmann constant, $m_{0} m_{r}$ is the effective mass of electrons, and $g$ is a degeneration factor (typically 1). Given a temperature dependent time constants distribution, such as the one mapped in Fig. 5b, each $E_{a}$ and $\sigma_{c}$ can be uniquely extracted as:

13 $$ (E_{a,i} , \sigma_{c,i} ,A_{i} ) \leftrightarrow \left( {\begin{array}{*{20}c} {\tau_{i1} } & {T_{1} } & {A_{i} } \\ {\tau_{i2} } & {T_{2} } & {A_{i} } \\ {\tau_{i3} } & {T_{3} } & {A_{i} } \\ \end{array} } \right) $$

The results of this analysis, applied to GaN HEMTs, is shown in Fig. 4c,d. The two distributions show the values of activation energy and capture cross section for each of the traps responsible for the stretched exponential process. Remarkably, the extracted $E_{a}$ distribution appears to be consistent with the experimental results reported by Stradiotto et al*.*^14^ where, SiN/AlGaN/GaN structure is used.

## TCAD simulation

To demonstrate the correctness of the of trap-state mapping procedure, we used the obtained trap-parameters as input for TCAD simulations and compared the simulated recovery traces with the experimental ones. Specifically, the recovery transients were simulated in Sentaurus TCAD by including the obtained distribution of traps (with the related $E_{a,i}$ and $\sigma_{c,i}$) at the passivation/AlGaN interface.

Details on the device structure and the employed TCAD model can be found in^36^. Traps are defined according to the activation energy and cross section distributions obtained via trap-state mapping, and are uniformly distributed at the surface as acceptors (see Fig. 5a). In addition, surface donors (0.8 eV from CB), are equally distributed in the access region^37^. The simulation, solving for standard drift–diffusion equations, consists in forcing all acceptors to be occupied before starting the recovery ($t = 0$), when their occupation state is then unfrozen. The simulation then considers that in $t = 0$ all acceptors are filled with electrons, and mimics the recovery transient when 0 V is applied at the contacts, thus accounting exclusively for thermal-induced recovery.

Snapshots of the device are saved during the recovery phase and are fed as input to a separate script where the device is biased in the linear region with $V_{G} = 4$ V, $V_{D} = 0.4$ V, in order to emulate the experimental recovery traces.

The simulated recovery traces, reported in Fig. 5b, are normalized to the nominal current and display excellent matching with the experimental ones. This demonstrates that the trap distribution obtained by trap-state mapping can effectively reproduce the dynamic behavior of the devices, even in presence of non-exponential recovery traces.

The interface traps density is used as fitting parameter in order to match the initial current degradation after 10 µs. As observable in Fig. 5c,d, the electron emission from SA during recovery leads to a decrease of the electric field in the AlGaN barrier and the repopulation of the 2DEG density in the channel.

## Conclusion

We have proposed a general methodology for mapping the properties (activation energy, cross sections) of a distribution of surface/interface states in GaN-based electronic devices. The properties of the traps are extracted through a mathematical approach, based on inverse Laplace transform. The developed methodology is used to univocally extract the properties of distributions of traps filled during hot-electron stress in GaN-based transistors.

To prove the validity and usefulness of the model, the extracted map distributions are used as input for TCAD simulations. The results obtained by TCAD closely match the experimental transient curves, thus confirming the effectiveness of the developed technique. The proposed methodology is generic and can be extended to other semiconductors for high-speed/high-voltage electronics, including AlN, gallium oxide, and diamond, thus representing a powerful tool for quantitatively assessing and modeling the properties of trap states.

## Data Availability

The data that support the findings of this study are available from the corresponding author upon reasonable request.
